# Editorial: Therapeutic potential of cannabinoids: from health to disease

**DOI:** 10.3389/fnins.2025.1565790

**Published:** 2025-02-19

**Authors:** Évila Lopes Salles, Babak Baban, Xu Qin, Valdemar Paffaro

**Affiliations:** ^1^Oral Biology, Dental College of Georgia, Augusta University, Augusta, GA, United States; ^2^Center for Excellence in Research, Scholarship, and Innovation (CERSI), Dental College of Georgia, Augusta University, Augusta, GA, United States; ^3^Department of Stomatology, Tongji Hospital, Tongji Medical College, Huazhong University of Science and Technology, Wuhan, China; ^4^Department of Cellular and Developmental Biology, Institute of Biomedical Sciences, Federal University of Alfenas, Alfenas, Brazil

**Keywords:** medicinal cannabis, cannabinoids, cannabidiol (CBD), tetrahydrocannabinol (THC), medicinal cannabis history

Cannabinoid history is deeply connected with human culture, medicine, and pharmacology, tracing back thousands of years. According to palaeobotanical evidence, cannabis' early uses are dated about 12,000 years ago in two domestication centers situated in Europe and Central Asia. Through the years cannabis spread to the Middle East and Africa (2000–500 year BP) and then to the Americas from Europe (1545–1800 CE) and Asia (1800–1945) (Warf, 2014; Crocq, 2020; Rull, 2022). Through these years cannabis varied uses and was associated with alleviating illnesses (Mirzaei et al., 2020), rituals and recreational practices (Kovalchuk et al., 2020; Mirzaei et al., 2020), and as a source of fiber for textiles, food, and oil (Van Bakel et al., 2011; Salami et al., 2020).

In its recent history, stigma, and prohibition, particularly during the 20th century, have complicated cannabis research and use, despite its importance (Russo, 2007; Maida and Daeninck, 2016). The identification of cannabinoids, especially delta-9-tetrahydrocannabinol (THC) and cannabidiol (CBD), represented a pivotal moment in cannabinoid research. This was particularly significant following the discovery of the endocannabinoid system in the 1990s, which established a biological foundation for comprehending the effects of these compounds (Zuardi, 2006; Appendino, 2020).

Many countries' recent legalization movement has sparked interest in its therapeutic uses, which has revived scientific research and public acceptance. Research on the active elements of cannabis has strengthened the present view of marijuana as a medicinal tool. In the past decades, the therapeutic potential of cannabinoids has been explored in several research areas.

In the present Research Topic “*Therapeutic Potential of Cannabinoids: From Health to Disease*,” the journal aimed to shed light on the local and systemic effects of cannabis and cannabinoids on cognitive function, and their implications in neurodegenerative and age-related diseases. In the manuscript “The effects of standardized cannabis products in healthy volunteers and patients: a systematic literature review,” Leen et al. review the effects of standardized cannabis products, especially those made by Bedrocan, on patients and healthy volunteers. The authors emphasize how crucial consistent cannabis formulations are for successful therapy, pointing to dose-dependent acute effects in healthy volunteers, like anxiety and impaired cognition. With few minor adverse effects, medicinal cannabis has been shown to help individuals with chronic pain. In order to evaluate cannabis dosage, composition, and other variables including age and gender to determine safety and efficacy, the review recommends additional randomized clinical trials.

Yndart Arias et al. also discuss the importance of standardized formulations and consistent dosing to ensure reliability. The authors discuss the need for more thorough research to determine the best dosage, formulations, and therapeutic efficacy for neurocognitive disorders while reviewing randomized trials assessing cannabidiol (CBD) as a possible treatment for cognitive impairments. By demonstrating the advantages of CBD for anxiety and cognition and emphasizing the need for more carefully planned research to confirm its use in treating neurocognitive impairments like Alzheimer's disease, this manuscript has the potential to further our understanding of CBD as a therapeutic option for cognitive disorders.

Also focusing on clinical research, in their review “*Implications for blinding in clinical trials with THC-containing cannabinoids based on the CANNA-TICS trial*,” Müller-Vahl et al. discuss the issue of unblinding clinical trials using THC-containing cannabinoids, specifically examining the unblinding rates in the CANNA-TICS trial. It states that 17% of individuals unblinded themselves on purpose or by mistake, and that some participants were not aware that they could do so. Due to the potential impact of growing public awareness of THC testing on trial integrity, the study emphasizes the significance of addressing unblinding concerns in subsequent randomized controlled trials utilizing THC-containing cannabinoids.

Using an animal model, Melkumyan et al. investigated how mice's anxiety-like behaviors and neuroimmune function were affected by cannabidiol (CBD) and a CBD: THC (3:1) mixture during alcohol withdrawal. It reveals that whereas CBD and CBD: THC enhanced anxiety during the first 4 h of withdrawal, CBD by itself decreased anxiety throughout the next 24 h. According to the study, using cannabis during alcohol withdrawal may have different impacts on anxiety depending on when it is used. These effects may be related to modifications in the central amygdala's neuroimmune function. This manuscript contributes to understanding the potential therapeutic effects of cannabinoids, specifically CBD and CBD: THC, on neuroimmune function modulation in the central amygdala. These results may help guide future studies and therapeutic strategies for treating alcohol withdrawal and related anxiety.

The present Research Topic offers a comprehensive perspective on cannabis research and highlights that effectiveness of medicinal cannabis treatments and patient results are largely dependent on the dosage and formulation evaluation of cannabinoids. Accurate dosage reduces the possibility of side effects while guaranteeing that patients get the right quantity of cannabinoids to produce therapeutic benefits. Inhalants, oils, and foods are examples of formulations that alter the bioavailability and commencement of action, which can have a substantial impact on clinical results (Bhandari et al., 2024). Moreover, personalized dosing based on factors like patient age, weight, metabolism, and the specific condition being treated can enhance treatment efficacy.

Without proper assessment and standardization, variations in dosing and formulation can lead to inconsistent results and undermine the potential benefits of medical cannabis for patients. Research on these issues is essential to establish clear guidelines and best practices for cannabinoid use in clinical settings. Investigating optimal dosing regimens and formulation types can provide evidence-based recommendations, ensuring patients receive safe and effective treatments. Furthermore, research can help identify the most suitable formulations for various conditions, refine therapeutic protocols, and reduce the risks of misuse or adverse effects. Understanding the entourage effect, the phenomenon where cannabinoids work synergistically with other compounds in the cannabis plant, also plays a crucial role in maximizing therapeutic outcomes (Baban et al., 2021). Understanding how the entourage effect boosts the effectiveness of cannabinoids may help improve treatment plans and provide patients with more individualized and thorough care. In the end, a better comprehension of these factors will promote the therapeutic use of medical cannabis, enhancing patient outcomes and leading to more potent therapies.

The field of cannabinoid research is characterized by considerable deficiencies in mechanistic understanding. Cannabinoids, for instance, have shown promise in treating pain and stiffness, but little is understood about the underlying molecular pathways (Lu and Anderson, 2017; Cintosun et al., 2020). The same is seen in cancer, an area in which cannabinoids have shown *in vitro* and *in vivo* effects on cell proliferation (Kalenderoglou et al., 2017; Kim et al., 2019) and tumor progression (Salles et al., 2023; Wang et al., 2024). However, we still need to understand these mechanisms in different types of cancer at the molecular level.

In summary, cannabinoids' history is intimately linked to human culture and medicine, having developed from their traditional ceremonial, recreational, and therapeutic applications to their status as a major study area with great promise. Even though these chemicals show great potential, there are still a lot of unanswered questions regarding their underlying processes and best uses. To resolve these scientific ambiguities and fully realize the medicinal promise of cannabis, future research—including well-planned clinical trials—is crucial. With more research, cannabis could become an essential tool for the treatment and prevention of a wide array of health conditions.
